# Genetic analysis of HIV-1 Circulating Recombinant Form 02_AG, B and C subtype-specific envelope sequences from Northern India and their predicted co-receptor usage

**DOI:** 10.1186/1742-6405-6-28

**Published:** 2009-12-03

**Authors:** Ujjwal Neogi, Vikas Sood, Arpita Chowdhury, Shukla Das, Vishnampettai G Ramachandran, Vijesh K Sreedhar, Ajay Wanchu, Nilanjana Ghosh, Akhil C Banerjea

**Affiliations:** 1 Virology Laboratory, National Institute of Immunology, Aruna Asaf Ali Marg, New Delhi 110067 India; 2 Microbiology Department, UCMS and GTB Hospital, Delhi 110095 India; 3 Department of Internal Medicine, PGIMER, Sector 12, Chandigarh 160012 India

## Abstract

HIV-1 epidemic in India is largely driven by subtype C but other subtypes or recombinants have also been reported from several states of India. This is mainly due to the co-circulation of other genetic subtypes that potentially can recombine to generate recombinant/mosaic genomes. In this study, we report detail genetic characterization of HIV-1 envelope sequences from North India (Delhi and neighboring regions). Six of 13 were related to subtype C, one B and the rest six showed relatedness with CRF02_AG strain. The subtype C possessed the highly conserved GPGQ motif but subtype B possessed the GPGR motif in the V3 loop as observed earlier. While most of the sequences suggested CCR5 co-receptor usage, one subtype C sample clearly indicated CXCR4 usage. A successful mother to child transmission was established in two pairs. Thus, co-circulation of multiple subtypes (B and C) and the recombinant CRF02_AG strains in North India suggests a rapidly evolving scenario of HIV-1 epidemic in this region with impact on vaccine formulation. Since this is the first report of CRF02_AG envelope from India, it will be important to monitor the spread of this strain and its impact on HIV-1 transmission in India.

## Introduction

HIV-1 displays a tremendous amount of genetic diversity. The binding of the HIV-1 to host cells is mediated by envelope glycoprotein. When the HIV-1 envelope protein binds to its primary receptor CD4, it undergoes conformational changes and it then binds to one of the coreceptors (chemokine receptor CCR5, CXCR4 or others) via its V3 loop. This tri-molecular interaction leads to the viral membrane fusion [1]. HIV-1 envelope is composed of relatively conserved (C1 to C5) and variable regions (V1 to V5). The V3 region elicits neutralizing antibodies and also govern co-receptor usage [1,2]. Replacements in the V3 region with basic amino acids are associated with CXCR4 usage [2,3]. Subtypes A and C usually contain a highly conserved GPGQ amino acid motif, while GPGR is the predominant motif in the V3 loop of subtype B envelopes [4,5]. Mutational patterns in the V3 loop region are likely to be of clinical significance as they can influence their susceptibility to known CCR5 inhibitors. Although all HIV-1 genetic subtypes originated in Africa, it is not fully understood how certain subtypes dominate different regions of the world. For e.g. subtype B predominates in US and UK but subtype C is predominant in India, some parts of Asia and Africa [6].

It is fairly well established that HIV-1 that uses CCR5 chemokine receptor (R5-tropic) is transmitted preferentially than the ones that use CXCR4 chemokine receptor [7]. Individuals with a 32 bp deletion in the CCR5 open reading frame (ORF) are largely protected against HIV-1 infection [7-9]. Approximately 50% of HIV-1 subtype B infected individuals show HIV-1 co-receptor switch from CCR5 to CXCR4 which is associated with rapid progression of HIV/AIDS [10]. This is observed mainly in US and UK where subtype B predominates. However, in India, where subtype C predominates, the coreceptor switch has not been observed [11]. Replacements of charged amino acids within the V3 region are known to alter the co-receptor usage [2,3,12]. Genetic variations in the subtype C HIV-1 envelope sequences have recently been reported from Southern India with some strains exhibiting multiple co-receptor usage, including CXCR4 chemokine receptor, present predominantly on T-helper lymphocytes [13,14]. It is noteworthy that we recently reported novel B/C LTR [15] and Vpr B/C/D sequences from North India [16].

Given the large size of India, and with increasing global travel, it is likely that subtypes other than B may also co-circulate, creating an ideal situation for the formation of recombinants. With this in mind, we genetically characterized the HIV-1 envelope sequences from HIV-1 infected individuals from Northern India and report the presence of HIV-1 CRF02_AG for the first time.

## Methods

### Genomic DNA isolation and Polymerase chain reaction

Genomic DNA was isolated from fresh peripheral blood collected in EDTA using a kit from Qiagen (QIAamp Blood Minikit) as described before by us [8,9]. All requisite ethical clearances were obtained before initiating this study. All the polymerase chain reactions (PCRs) were performed with high fidelity Taq DNA polymerase (Ex-Taq, Takara, Japan) using the following primers:

Forward primer: 5'-ATGGGATCAAAGCCTAAAGCCATGTG

Reverse primer: 5'-AGTGCTTCCTGCTGCTCCCAAGAACCCAAG

Approximately 1.25 Kb DNA fragment corresponding to V1 to V5 region was amplified initially. Thereafter, 700 bp fragment (V3 to V5) was amplified using two internal sets of primers with following sequences:

Forward primer: CTGTTAAATGGCAGTCTAGC

Reverse primer: CACTTCTCCAATTGTCCCTCA

The cycling conditions for amplifying both the fragments were: 35 cycles at 98°C for 15 sec, 55°C for 30 sec and 72°C for 1 min with a final extension at 72°C for 10 min. PCR-amplified DNA was cloned into pGem-T expression vector (Promega Biotech. WI, USA) and sequenced in both directions using T7 and SP6-specific primers. The sequence from one representative clone from each sample was used to carry out phylogenetic analysis and sequence comparisons. The final concentration of MgCl_2 _was 20 mM for both the PCRs. Mother and child samples were processed separately to avoid cross contamination.

### Patient population and genetic analysis

**Figure 1:**
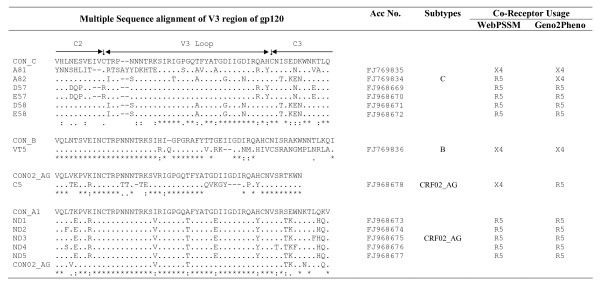
**HIV-1 envelope sequence comparison and coreceptor usage**. HIV-1 envelope gene was amplified from infected individuals and subjected to sequencing as described in the text. Only the V3 loop region sequences with short flanking constant regions are shown with their accession numbers, their subtype assignment and possible co-receptor usage. Dots in the sequence indicate identity with consensus C, B, 02_AG and A sequences; asterisk indicates identical amino acids; single dot at the bottom of four groups of samples represents semi-conserved substitution of amino acids and double dots represent conserved substitution. Subtypes were determined using Viral Genotyping Tool, REGA Subtyping Tool and RIP 3.0 with maximum blast identity.

We carried out genetic analysis of 13 HIV-1 envelope sequences from Northern India. Nine unrelated and 2 mother-child pairs (Pair 1, D & E 57 and Pair 2, D & E 58) were selected randomly from two locations (one from GTB Hospital, Delhi - Samples ND1 to 5, all from commercial sex workers-CSW) and the rest were from Punjab/Haryana region. Primers were designed to carry out nested PCR as described earlier. It is noteworthy that we were unable to amplify envelope sequences from several samples which may be due to extreme genetic variability and therefore difficult to draw conclusions about the frequency of any genetic subtype from this study. Alternatively, since most of the HIV-1 infected individuals were on antiretrovirals, the amounts of proviral DNA may have been too small to amplify. Sequences were compared with reference strains (figure 1) (Los Almos-http://www.hiv.lanl.gov). At least 4 independent clones were analyzed from each sample and only one representative clone from each sample was genetically analyzed. Multiple sequence analysis was performed in ClustalW 1.8.3 obtained from DNA data bank of Japan (DDBJ) website http://clustalw.ddbj.nig.ac.jp/top-e.html. The phylogenetic analysis was carried out using MEGAA 4.1 (beta) software. Genotyping was carried out using viral genotyping tools located at NCBI http://www.ncbi.nlm.nih.gov/projects/genotyping/formpage.cgi, REGA subtyping tool ver 2.0 http://www.bioafrica.net/subtypetool/html and Recombination Identification Program (RIP) 3.0. http://www.hiv.lanl.gov/content/sequence/RIP/RIP.html. Potential N-glycosylation sites were calculated using N-GlycoSite program http://www.hiv.lanl.gov/content/sequence/GLYCOSITE/glycosite.html.

## Results and discussion

**Figure 2:**
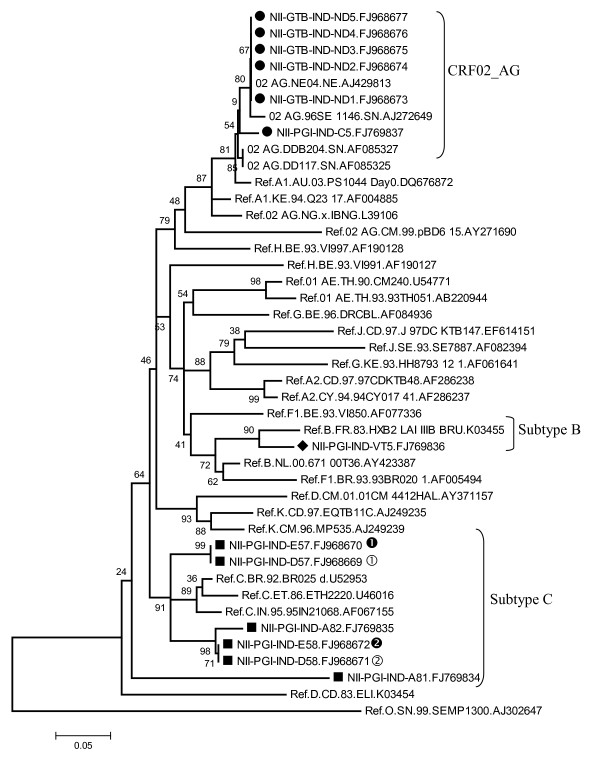
**Phylogenetic analysis of the HIV-1 envelope sequences from North India**. All the reference sequences from M-group & outlier group were retrieved from Los Almos Data Base and used for constructing neighbor joining phylogenetic tree. Indian CRF02_AG strains are represented as 'Black Circles'; 'Black Square' for subtype C; and 'Black Diamond' as subtype B. The evolutionary history was created using Neighbor-Joining Method in MEGA4. Similar evolutionary pattern was detected when Maximum Likelihood and UPGMA methods were used (data not shown). Mother-child pairs were shown by filled and empty circles by numbers (1 and 2). Empty circles denote maternal envelope sequences while filled circles denote infant envelope sequences.

All of the HIV-1 infected individuals were infected through heterosexual route (except mother-child pair) and their CD4 count varied from 120 - 150 (sample A81 & 82) and between 400-500 (D57 and D58). Most of them were under 1^st ^line of antiretroviral treatment. The GPGQ motif present in the middle of the V3 loop was conserved among all subtype C and CRF_02 AG strains. Remarkably 5 of subtype C samples showed conservation of A residue just downstream of GPGQ motif (not observed in consensus C) and 4 of them showed H to Y change just prior to the second cysteine of the V3 region (figure 1). The subtype B sample (VT5) possessed the GPGR amino acid motif at the crown of the V3 loop as expected. It is noteworthy that we recently reported novel mosaic B/C HIV-1 LTR and B/C/D recombinant *Vpr *structures from the same region of India (Punjab/Haryana region) [15,16]. Group M subtype reference sequences along with outlier sequences were downloaded from Los Almos HIV data base. The sequences were subjected to various genetic subtyping tools (Phylogenetic Analysis, RIP 3.0, Viral Genotyping Tools and Rega Subtyping). This analysis indicated that 6 of 13 were related to subtype C, one B and the rest 6 showed resemblance with CRF02_AG strain (figure 2). Successful mother-to-child transmission was detected in both the pairs (Bootstrap value 99 in pair 1 and 71 in Pair 2) as judged by high bootstrap value (figure 2). It is noteworthy that no changes in the V3 sequences were observed in both the mother-infant pairs. Maximum intra-patient proviral diversity was observed in two samples (A81 and C5) (manuscript under preparation). It was reported earlier that subtype determination based on phylogenetic analysis should also be confirmed by using other tools or signature sequences present in V3 region [17]. Representative subtype sequences identified by RIP 3.0 program are given (additional file 1). Each curve is a comparison between the envelope regions being analyzed (query- as indicated at the top of each square) and multiple reference sequences downloaded from the data bank. Using this kind of analysis, HXB2 (panel A) and an isolate with an accession number FJ769836 (panel B), were identified as subtype B; isolate FJ968673 as CRF_02AG (panel C) and isolate with an accession number FJ968672 as subtype C.

The most remarkable finding was the predominance of CRF02_AG strain among the unrelated commercial sex workers (CSWs) from Delhi (Capital of India) region. All the isolates from Punjab/Haryana region showed relatedness with consensus C. This recombinant form is predominantly found in Africa (Cote Divoire, Mali, Senegal, Ghana and Cameroon etc.) followed by Korea, Spain and France. The potential glycosylation sites present in V3 to V5 region varied from 7 (A81) to 15 (ND5 (data not shown). This is important because in some instances hypo-glycosylated forms of envelope have been associated with better transmission and in their ability to interact with neutralizing antibody [18].

It was remarkable that sample A81 clearly showed CXCR4 coreceptor usage by both the programs (WebPSSM and Geno2Pheno) designed to predict HIV-1 coreceptor usage. This is important because earlier studies with Indian subtype C envelope showed exclusive use of CCR5 co-receptor [11]. It is important to note that Samples A82 and C5 showed discrepancy in their predicted coreceptor usage and this is because the two programs use different parameters [19,20].

Successful transmission of virus (judged by high bootstrap values) was observed in both the mother-child pair samples. It is important to study the functional implications of the changes in the viral gene sequences between mother-infant pairs to understand the molecular basis of successful transmission [21]. VT5 (subtype B) sample, as expected, showed CXCR4 usage and all of the CRF02_AG strains showed CCR5 usage.

In summary, we show for the first time presence and transmission of CRF02_AG HIV-1 strain in India (Delhi - Capital of India) and presence of subtypes B and C in North India. These observations will impact on the T-cell epitope based vaccine. The existence of multiple HIV-1 genetic subtypes in this region is likely to generate novel and complex recombinants.

## Competing interests

The authors declare that they have no competing interests.

## Authors' contributions

UN, VS, NG and AC carried out the experiments. SD, VGR, VKS, AW were responsible for providing the blood samples and their clinical characteristics. ACB is the principal investigator responsible for designing the work and writing the manuscript. VS and UN contributed equally to this work.

## Supplementary Material

Additional file 1 **Identification of HIV-1 subtypes**. RIP tool available in Los Alamos HIV Database was used to type four representative query sequences (HXB2 - panel A; FJ769836 - panel B; FJ968673 - panel C and FJ968672 - panel D) as indicated at the top of each square. Similarity of the sequences was compared with various subtypes with a window size 400 bp having significant threshold (0.9). It is noteworthy that HXB2 (Accession no. K03455, panel A) and FJ769836 (NII-PGI-IND-VT5) (Panel B) were identified as subtype B (lemon green); FJ968673 (NII-GTB-IND-ND1) as 02_AG (dark green, panel C) and FJ968672 (NII-PGI-IND-E58) as subtype C (blue, panel D). Click here for file

## References

[B1] Dragic T Litwin V Allaway GP Martin SR Huang KA Nagashima A Cayanan C Maddon PJ Koup RA Moore JP Paxton WA HIV-1 entry into CD+ cells is mediated by the chemokine receptor CC-CKR-5 Nature 1996 381 667 673 10.1038/381667a0 8649512

[B2] Hoffman NG Seillier-Moiseiwitsch F Ahn J Walker JM Swanstrom Variability in the human immunodeficiency virus type 1 gp120 Env protein linked to phenotype associated changes in the V3 loop J Virol 2002 76 3852 3864 10.1128/JVI.76.8.3852-3864.2002 11907225 PMC136063

[B3] Milch LB Margolin B Swanstrom R V3 loop of the immunodeficiency virus type 1 Env protein: interpreting sequence variability J Virol 1993 67 5623 5634 8350415 10.1128/jvi.67.9.5623-5634.1993 PMC237966

[B4] Korber BT MacInnes K Smith RF Myers G Mutational trends in V3 loop protein sequences observed in different genetic lineages of human immunodeficiency virus type 1 J Virol 1994 68 6730 6744 8084005 10.1128/jvi.68.10.6730-6744.1994 PMC237094

[B5] Stanfield RL Gorny MK Zolla-Pazner S Wilson IA Crystal structures of human immunodeficiency virus type 1 (HIV-1) neutralizing antibody 2219 in complex with three different V3 peptides reveal a new binding mode for HIV-1 cross-reactivity J Virol 2006 80 6093 6105 10.1128/JVI.00205-06 16731948 PMC1472588

[B6] Hemelaar J Gouws E Ghys PD Osmanov S Global and regional distribution of HIV-1 genetic subtypes and recombinants in 2004 AIDS 2004 20 W13 23 10.1097/01.aids.0000247564.73009.bc 17053344

[B7] Berger EA Murphy PM Farber JM Chemokine receptors as HIV-1 coreceptors: Roles in viral entry, tropism, and disease Annu Rev Immunol 1999 17 657 700 10.1146/annurev.immunol.17.1.657 10358771

[B8] Husain S Goila R Shahi S Banerjea AC First report of a healthy Indian heterozygous for Δ32 mutant of HIV-1 coreceptor-CCR5 gene Gene 1998 207 141 147 10.1016/S0378-1119(97)00617-3 9511755

[B9] Husain S Goila R Shahi S Banerjea AC Inheritance pattern of mutant human immunodeficiency virus type 1 coreceptor gene in an Indian family J Hum Virol 1998 1 187 192 10195241

[B10] Connor RI Sheridan KE Ceradini D Choe S landau NR Change in coreceptor usage correlates with disease progression in HIV-1 infected individuals J Exp Med 1997 185 621 628 10.1084/jem.185.4.621 9034141 PMC2196142

[B11] Cecilia D Kulkarni SS Tripathy SP Gangakhedkar RR Paranjape RS Gadkari DA Absence of coreceptor switch with disease progression in human immunodeficiency virus infection in India Virology 2000 271 253 258 10.1006/viro.2000.0297 10860879

[B12] Pastore C Nedelle R Ramos A Pontow S Ratner L Mosier DE Human immunodeficiency virus type 1 coreceptor switching: V1/V2 gain-of-fitness mutations compensate for V3 loss-of fitness mutations J Virol 2006 80 750 758 10.1128/JVI.80.2.750-758.2006 16378977 PMC1346864

[B13] Dash PK Sidappa NB Mangaiarkarasi A Mahendarkar AV Roshan P Anand KK Mahadevan A Satishchandra P Shankar SK Prasad VR Ranga U Exceptional molecular and coreceptor-requirement properties of molecular clones isolated from an Human Immunodeficiency Virus type-1 subtype C infection Retrovirology 2008 5 25 10.1186/1742-4690-5-25 18328091 PMC2292743

[B14] Gharu L Ringe R Pandey S Paranjape R Bhattacharya J HIV-1 clade C env clones obtained from an Indian patient exhibiting expanded coreceptor tropism are presented with naturally occurring unusual amino acid substitutions in V3 loop Virus Res 2009 144 306 314 10.1016/j.virusres.2009.04.020 19409946

[B15] Neogi U Sood V Goel N Wanchu A Banerjea AC Novel HIV-1 long terminal repeat (LTR) sequences of subtype B and mosaic intersubtype B/C recombinants in North India Arch Virol 2008 153 1961 1966 10.1007/s00705-008-0210-y 18818865

[B16] Bano AS Sood V Neogi U Goel N Kuttiat VS Wanchu A Banerjea AC Genetic and functional characterization of HIV-1 VprC variants from North India: Presence of unique recombinants with mosaic genomes from B, C and D subtypes within the ORF of Vpr J Gen Virol 2009 90 2768 2776 10.1099/vir.0.011080-0 19605589

[B17] Vasil S Thakallapally R Korber BT Foley BT Korber B, Kuiken CL, Foley B, Hahn B, McCutchan F, Mellors JW, Sodroski J Global Variation in the HIV-1 V3 Region Human Retroviruses and AIDS 1998 1998 Theoretical Biology and Biophysics Group, Los Alamos National Laboratory, Los Alamos, NM III-118 129

[B18] Derdeyn CA Decker JM Bibollet-Ruche F Mokili JL Muldoon M Denham SA Heil ML Kasolo F Musonda R Hahn BH Shaw GM Korber BT Allen S Hunter E Envelope constrained neutralization-sensitive HIV-1 after heterosexual transmission Science 2004 303 2019 2022 10.1126/science.1093137 15044802

[B19] Sing T Low AJ Beerenwinkel N Sander O Cheung PK Domingues FS Büch J Däumer M Kaiser R Lengauer T Harrigan PR Predicting HIV co-receptor usage based on genetic and clinical covariates Antivir Ther 2007 12 1096 1106 18018768

[B20] Jensen MA Li FS Van't Wout AB Nickle DC Shriner D He HX McLaughlin S Shankarappa R Margolick JB Mullins JI Improved coreceptor usage prediction and genotypic monitoring of R5-to-X4 transition motif analysis of HIV-1 env V3 loop sequences J Virol 2003 77 13376 13388 10.1128/JVI.77.24.13376-13388.2003 14645592 PMC296044

[B21] Mehta R Sundaravaradan V Ahmad N Mutations generated in human immunodeficiency virus type 1 long terminal repeat during vertical transmission correlate with viral gene expression Virology 2008 317 109 118 10.1016/j.virol.2008.01.048 PMC2430019 18313715

