# Supplementary figures and images for: Genetic analysis of HIV-1 Circulating Recombinant Form 02_AG, B and C subtype-specific envelope sequences from Northern India and their predicted co-receptor usage
Source: AIDS Res Ther. 2009 Dec 3;6:28. doi: 10.1186/1742-6405-6-28 (PMC2794860; doi:10.1186/1742-6405-6-28)

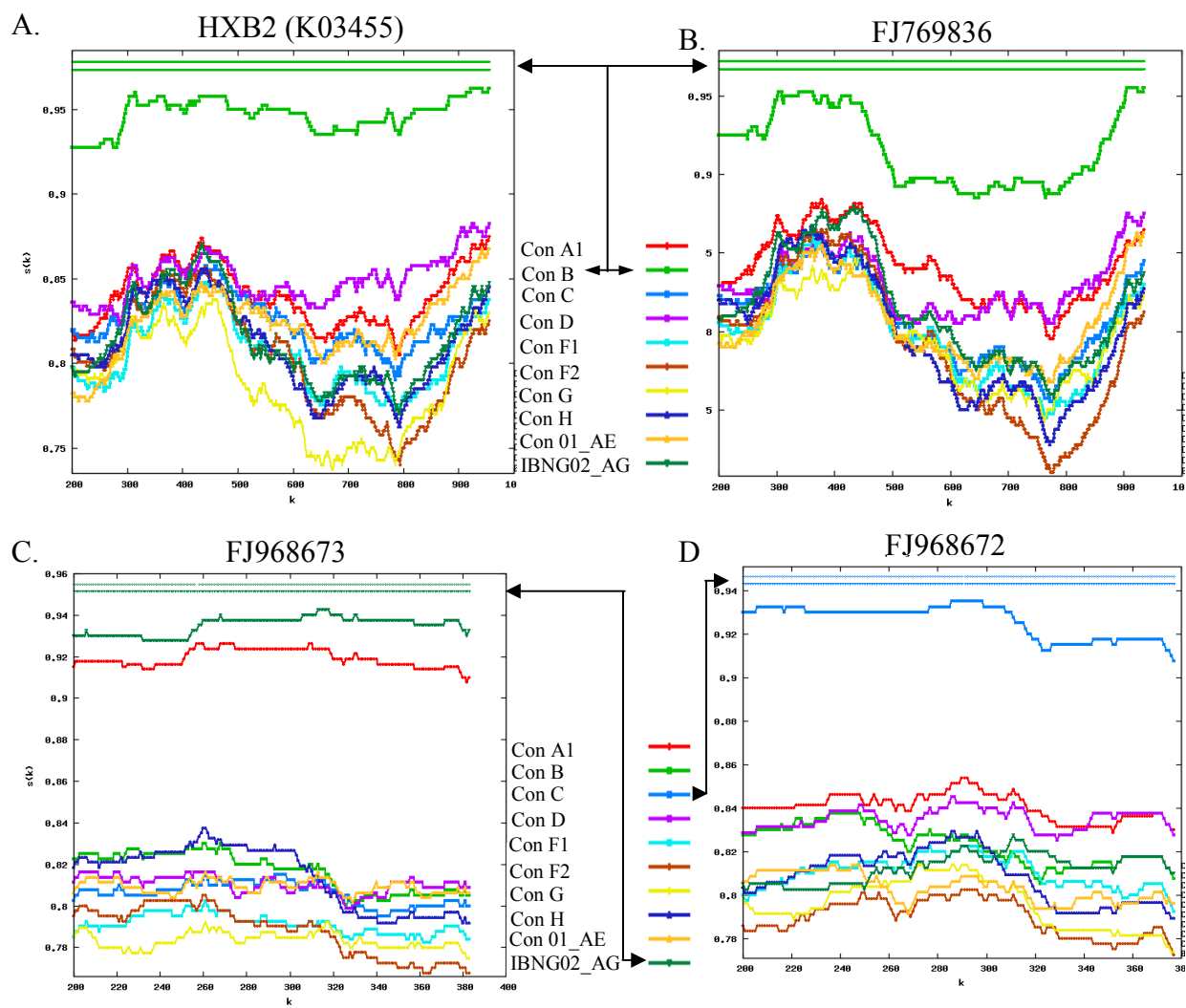

Supplement: Additional file 1 — Identification of HIV-1 subtypes. RIP tool available in Los Alamos HIV Database was used to type four representative query sequences (HXB2 - panel A; FJ769836 - panel B; FJ968673 - panel C and FJ968672 - panel D) as indicated at the top of each square. Similarity of the sequences was compared with various subtypes with a window size 400 bp having significant threshold (0.9). It is noteworthy that HXB2 (Accession no. K03455, panel A) and FJ769836 (NII-PGI-IND-VT5) (Panel B) were identified as subtype B (lemon green); FJ968673 (NII-GTB-IND-ND1) as 02_AG (dark green, panel C) and FJ968672 (NII-PGI-IND-E58) as subtype C (blue, panel D). [file 1742-6405-6-28-S1.PDF]
